# Finding New Genes for Non-Syndromic Hearing Loss through an In Silico Prioritization Study

**DOI:** 10.1371/journal.pone.0012742

**Published:** 2010-09-28

**Authors:** Matteo Accetturo, Teresa M. Creanza, Claudia Santoro, Giancarlo Tria, Antonio Giordano, Simone Battagliero, Antonella Vaccina, Gaetano Scioscia, Pietro Leo

**Affiliations:** GBS BAO Advanced Analytics Services and MBLab, IBM, Bari, Italy; King Abdullah University of Science and Technology, Saudi Arabia

## Abstract

At present, 51 genes are already known to be responsible for Non-Syndromic hereditary Hearing Loss (NSHL), but the knowledge of 121 NSHL-linked chromosomal regions brings to the hypothesis that a number of disease genes have still to be uncovered. To help scientists to find new NSHL genes, we built a gene-scoring system, integrating Gene Ontology, NCBI Gene and Map Viewer databases, which prioritizes the candidate genes according to their probability to cause NSHL. We defined a set of candidates and measured their functional similarity with respect to the disease gene set, computing a score (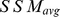) that relies on the assumption that functionally related genes might contribute to the same (disease) phenotype. A Kolmogorov-Smirnov test, comparing the pair-wise 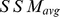 distribution on the disease gene set with the distribution on the remaining human genes, provided a statistical assessment of this assumption. We found at a *p-value* 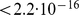 that the former pair-wise 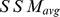 is greater than the latter, justifying a prioritization strategy based on the functional similarity of candidate genes respect to the disease gene set. A cross-validation test measured to what extent the 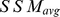 ranking for NSHL is different from a random ordering: adding 15% of the disease genes to the candidate gene set, the ranking of the disease genes in the first eight positions resulted statistically different from a hypergeometric distribution with a *p-value* 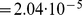 and a *power* 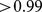. The twenty top-scored genes were finally examined to evaluate their possible involvement in NSHL. We found that half of them are known to be expressed in human inner ear or cochlea and are mainly involved in remodeling and organization of actin formation and maintenance of the cilia and the endocochlear potential. These findings strongly indicate that our metric was able to suggest excellent NSHL candidates to be screened in patients and controls for causative mutations.

## Introduction

Non Syndromic hereditary Hearing Loss (NSHL) is one of the most genetically heterogeneous disorders known. Indeed it can present an autosomal recessive, autosomal dominant, X-linked or mitochondrial pattern of inheritance; furthermore, mutations in the same gene may cause syndromic or non syndromic hearing loss, and recessive forms may be caused by a combination of two mutations in different genes from the same functional group [1].

Due to this tremendous genetic heterogeneity, the identification of genes and gene defects that affect the process of hearing is challenging [1]. At present 51 genes have been already identified to be responsible, if mutated, for this phenotype (see Table 1 for references); nevertheless not all these genes have been fully characterized. They usually are involved in the inner ear development or functionality, and their mutations generally cause hearing loss interfering in the process of the elaboration of sound.

**Table 1:** NSHL disease genes.

Gene Symbol	Locus Name	Chromosomal Location	References
DIAPH1	DFNA1	[5q31.3c]	[43]
GJB3	DFNA2	[1p34.3f]	[44]
KCNQ4	DFNA2	[1p34.2c]	[45]
GJB2	DFNA3/DFNB1A	[13q12.11a]	[2]
GJB6	DFNA3/DFNB1B	[13q12.11b]	[3], [46]
MYH14	DFNA4	[19q13.33c]	[47]
DFNA5	DFNA5	[7p15.3a]	[48]
WFS1	DFNA6/DFNA14	[4p16.1f]	[7], [8]
TECTA	DFNA8/DFNA12/DFNB21	[11q23.3h]	[49]
COCH	DFNA9	[14q12e]	[5]
EYA4	DFNA10	[6q23.2c]	[50]
MYO7A	DFNA11/DFNB2	[11q13.5c]	[51]
COL11A2	DFNA13/DFNB53	[6p21.32a]	[52]
POU4F3	DFNA15	[5q32d]	[53]
MYH9	DFNA17	[22q12.3d]	[54]
ACTG1	DFNA20/DFNA26	[17q25.3f]	[55], [56]
MYO6	DFNA22/DFNB37	[6q14.1a]	[57]
GRHL2	DFNA28	[8q22.3a-q22.3b]	[58]
TMC1	DFNA36/DFNB7/DFNB11	[9q21.13a]	[59]
CRYM	DFNA40	[16p12.2b]	[60]
CCDC50	DFNA44	[3q28d]	[61]
MYO1A	DFNA48	[12q13.3a]	[62]
KCNJ10	DFNA49	[1q23.2c]	[63]
MIRN96	DFNA50	[7q32.2a]	[64]
MYO15A	DFNB3	[17p11.2g-7p11.2f]	[65]
SLC26A4	DFNB4	[7q22.3c]	[4]
TMIE	DFNB6	[3p21.31a]	[66]
TMPRSS3	DFNB8/DFNB10	[21q22.3b]	[67]
OTOF	DFNB9	[2p23.3b]	[68]
CDH23	DFNB12	[10q22.1d-10q22.1e]	[69]
STRC	DFNB16	[15q15.3a]	[70]
USH1C	DFNB18	[11p15.1d]	[37], [38]
OTOA	DFNB22	[16p12.2a]	[71]
PCDH15	DFNB23	[10q21.1b-10q21.1c]	[72]
RDX	DFNB24	[11q22.3d]	[73]
TRIOBP	DFNB28	[22q13.1a]	[74], [75]
CLDN14	DFNB29	[21q22.13a]	[76]
MYO3A	DFNB30	[10p12.1b]	[77]
WHRN(DFNB31)	DFNB31	[9q32e]	[78]
ESRRB	DFNB35	[14q24.3c]	[79], [80]
ESPN	DFNB36	[1p36.31a]	[81]
HGF	DFNB39	[7q21.11c-q21.11d]	[82]
KIAA1199	DFNB48	[15q25.1b]	[83]
MARVELD2	DFNB49	[5q13.2a]	[84]
PJVK(DFNB59)	DFNB59	[2q31.2b]	[85]
SLC26A5	DFNB61	[7q22.1g]	[86]
LRTOMT	DFNB63	[11q13.4]	[87]
LHFPL5	DFNB66/DFNB67	[6p21.31b]	[40], [88], [89]
PRPS1	DFN2	[Xq22.3b]	[90]
POU3F4	DFN3	[Xq21.1d]	[6]
ATP2B2		[3p25.3b]	[91], [92]

GeneIDs are from NCBI Entrez Gene database; gene symbols correspond to the official gene names as provided by HUGO Gene Nomenclature Committee (HGNC); locus names have been inferred from literature; chromosomal locations are derived from the file cyto_gene.md downloaded from the NCBI Entrez Gene ftp site and references are relative to the articles where the gene association to NSHL was identified.

About 50% of cases of NSHL are due to mutations of GJB2, a gene coding for a gap-junction protein called connexin 26, involved in the cell-cell communication process. Another important gene responsible for NSHL is GJB6, belonging to the same family of GJB2 and adjacent to it. The identification of these two genes highlighted the role of connexins, and therefore of the cochlear gap-junction ion channels, in the auditory function [2], [3].

However the biology of hearing is extremely complex and many other different classes of genes are involved in NSHL. For instance, SLC26A4, associated with autosomal recessive NSHL [4] and Pendred syndrome, is a gene coding for pendrin, a chloride/iodide transporter; COCH, responsible for autosomal dominant non syndromic post-lingual with a progressive onset in adulthood [5], encodes for cochlin, a component of the extracellular matrix of the inner ear; POU3F4, responsible for an X-linked non syndromic progressive and profound sensorineural hearing loss [6], encodes for a transcription factor; while WFS1 associated with autosomal recessive Wolfram syndrome and autosomal dominant low frequency NSHL [7], [8], is a gene coding for the glycoprotein wolframin.

Moreover, several linkage studies over the years have shown that many chromosomal regions are involved in NSHL. At present 121 loci are known to be involved in this phenotype [9], and for many of them the genes causing NSHL have not been identified yet. Due to their often extremely large dimensions – they can even contain several hundreds of genes – it is not feasible to experimentally validate all the genes contained in each locus. In addition, some loci might contain more than one disease gene, as in the case of DFNA3 that harbors GJB2 and GJB6.

In this scenario, a bioinformatic approach to narrow down the list of possible candidate genes is an essential requirement in order to experimentally validate first those genes most likely associated with the disease.

Many strategies have been devised to address this issue, mostly sharing the common prioritization idea of ranking the candidate genes on the basis of their similarity with a set of training genes – genes already associated to the phenotype – relying on the main assumption that genes whose dysfunction contributes to a disease phenotype tend to be functionally related (see [10] and references within).

Quantifying the functional relatedness between two genes is not trivial; often existing information about gene function are exploited to infer functional relationships among genes. In this kind of approach an excellent means is provided by Gene Ontology (GO, The Gene Ontology Consortium, 2001) [11], which is the golden standard ontology in the field of genes and gene products.

Indeed one of the advantages of having genes annotated with GO terms is the possibility to compare them not only from a qualitative point of view (e.g. by searching for common terms with which they are annotated), but also by defining an explicit semantic similarity measure which reflects the closeness in meaning between the terms with which they are annotated [12], [13]. This semantic similarity measure gives in turn a measure of the functional similarity of the annotated gene products, as extensively discussed in Pesquita et al [12].

Briefly, when comparing two terms in an ontology, two main approaches are generally distinguished, the edge-based, which counts the edges in the graph path between two terms [14]–[18], and the node-based, which looks at the properties of the terms, their ancestors and descendants [19]–[25]. Most of the node-based similarity measures are functions of the information content (*IC*) of each term, and their most informative common ancestors [25]. *IC* is the amount of information a term contains, meaning that a term contains less information if it occurs very often; in this context the similarity between two terms is quantified looking at the amount of information they share. Very often gene products are annotated with multiple GO terms, in this case maximum [26], [27], average [13], [18], [28] or sum [29] of the GO term similarities may be taken as the gene similarity.

Here we define a new Semantic Similarity Measure (*SSM*) between gene products by directly extending to sets of concepts (the gene annotations) the Lin's idea [25] of quantifying the similarity between two concepts in an ontology. Our metric provides a measure of the functional similarity between two genes and its reliability is tested in this paper in the context of gene prioritization for NSHL. Indeed the overall aim of this paper is (i) to support researchers in search of new genes responsible for NSHL and (ii) provide indications about the main biological processes, molecular functions and cellular components to be explored to study NSHL, by defining a procedure to computationally prioritize candidate genes for their association with this phenotype. The availability of a good training gene set for NSHL – 51 genes already associated with this phenotype (disease genes) – allows to select new genes most likely responsible for this phenotype estimating their similarity with the disease gene set.

Finally we define a systematic and unbiased statistical assessment to validate the obtained results.

## Results

The candidate genes prioritized for NSHL in this study were selected as described in the Methods section. They were prioritized against all the genes already known to be responsible for NSHL (disease genes, see Methods section for details on their selection), according to a score which is function of the Semantic Similarity Measure (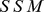) estimated for each candidate-disease gene pair. All candidate genes were ranked by computing the 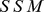 for each candidate-disease gene pair; the final score used for prioritizing each candidate was obtained as the mean of the scores estimated for that candidate against all the disease genes and was defined Semantic Similarity Measure Average (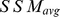).

### Validation of the 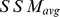 for NSHL gene prioritization

**Figure 1:**
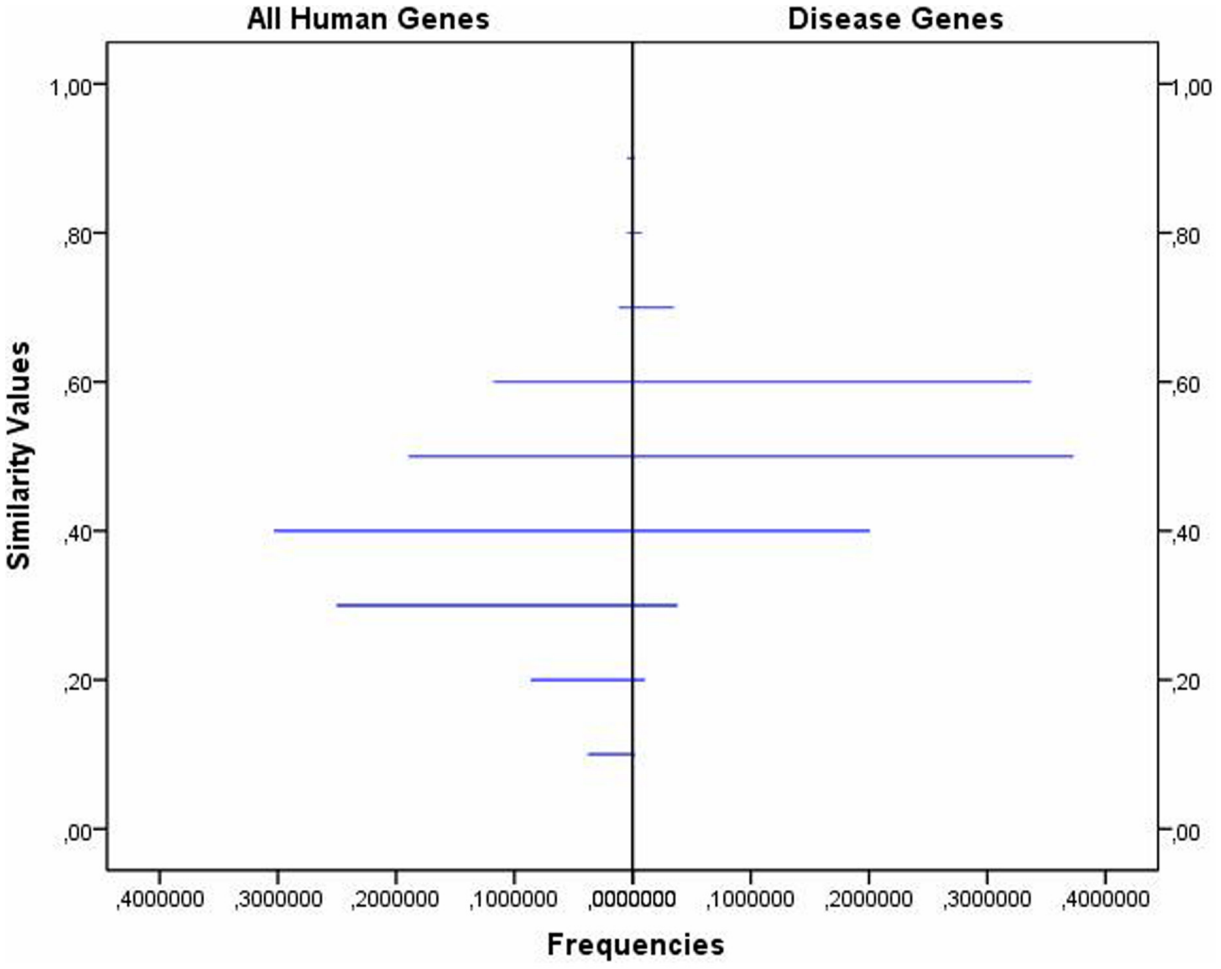
Similarity population pyramid. Back-to-back histograms showing the asymmetry in frequencies of SSM values (in 0.1 bin interval between 0 and 1) among gene pairs, for disease genes (on the right) and the entire human gene set (on the left).

Before being able to assert that the ranking produced by 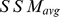 is worthy of attention and therefore evaluating it from a biological point of view, we wanted to evaluate two main aspects concerning our prioritization methodology. We first wanted to test whether the main hypothesis upon which this and most of the prioritization studies are based – genes whose dysfunction contributes to a disease phenotype tend to be functionally related – is quantifiable in terms of semantic similarity, especially in the particular case of NSHL, where the complexity of the hearing process and the complexity of the genetics of the disease both play an important role. Second aspect is whether our metric is able to catch this functional relatedness. To test these two aspects is equivalent to answer the following question: are the disease genes more functionally related than two generic human genes according to 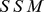
? A positive answer would yield a positive result for both aspects at the same time, implying that the more a candidate gene obtains a high 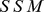 score respect to the disease gene set, the higher is its probability to cause NSHL when mutated. To address this issue, we estimated the pair-wise *SSM* distribution on the disease gene set, and compared it with the pair-wise *SSM* distribution estimated on the entire human gene set. In Figure 1 a population pyramid shows the pair-wise *SSM* distribution across the disease genes and All-human-genes sets in two back-to-back histograms. It provides the graphical evidence that the majority of the disease gene pairs assume 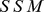 values in the range of 0.5–0.6, much greater than those assumed by the majority of all the remaining human genes (around 0.4). This clearly indicates that the NSHL genes are more functionally related in terms of 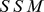 similarity than two generic human genes. In order to statistically support this result, we formulated the following test: the null hypothesis is that the pair-wise *SSM* distribution in the disease genes set is equal to the pair-wise *SSM* distribution in the All-human-genes set, while the alternative hypothesis is that the former is greater than the latter, i.e. the cdf (cumulative distribution function) of the former population is smaller than the cdf of the latter population. The test was performed using the bootstrap version of the Kolmogorov-Smirnov test (ks.boot), which allows ties and is included in the R package Matching [30]. We found a *p-value* 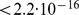, confirming the hypothesis that the disease genes are indeed more similar according to 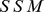 than two generic human genes. This evidence shows the ability of our metric in capturing the functional relatedness of NSHL genes respect to the rest of all human genes, justifying therefore a gene prioritization strategy for association with NSHL based on the 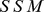 similarity of the candidate genes with respect to the disease gene set.

**Figure 2:**
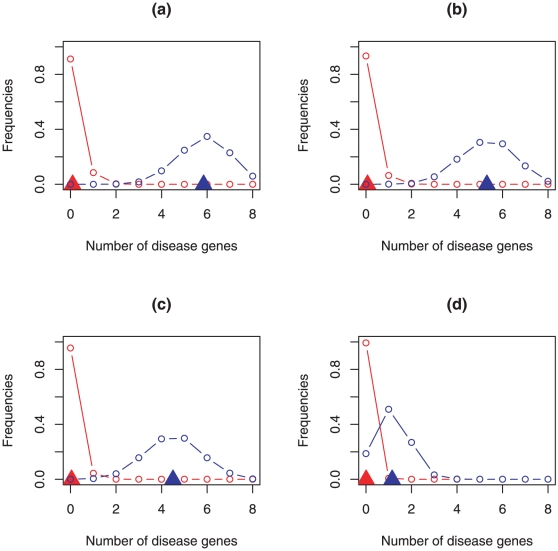
Cross-validation and hypergeometric distributions in case of (a)100, (b)75, (c)50, (d)8 window widths. In red the hypergeometric distributions with their expectation values (red filled triangles); in blue the distributions, estimated by cross-validation, of disease genes in the top-ranked genes with their mean values (blue filled triangles).

In order to validate the reliability of 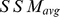 in ranking the candidate genes with respect to their probability to play a causative role in NSHL manifestation, we designed a specific cross-validation procedure that quantifies how much the ranking obtained with our metric differs from a random ordering of the candidate genes. Indeed, due to the specific context we are dealing with, i.e. the gene prioritization, we could not use the classical cross-validation procedure, we in fact added 15% of the disease genes randomly drawn for 10000 times from the disease gene set to the candidates, and counted each time the number of the diseases that fell in the top four windows of 100, 75, 50 and 8 genes. Here the candidate gene set was used exclusively to produce noise, as the positions of the candidates in the ranking were never evaluated during the cross-validation procedure. We in fact tested if the number of disease genes ranked in the top windows were significantly greater than expected when a random extraction of 100, 75, 50 and 8 genes was performed from the total (candidates plus 15% of disease genes) gene set. In Figure 2 we report the distributions obtained from the cross-validation procedure (in blue) applied to the four top windows. In this figure we compare these distributions with the hypergeometric ones (in red), which mimic the random extraction of 100, 75, 50 and 8 genes from the 8748 genes (8740 candidate genes plus 8 disease genes). In all four cases the two distributions are clearly distinct (i.e. the overlapping regions are small). Moreover the means of the distributions for the cross-validation (blue triangles in the figure) result always greater than the means of the hypergeometric distributions (red triangles in the figure). This confirms that the ranking computed by our gene scoring system is significantly different from a random ordering. This is equivalent to assert that our scoring system is able to put at the top of the ranking those genes which are functionally more related to the NSHL genes and thus, more likely, potentially to cause the disease when mutated. This evidence is statistically supported as the *p-value* and the *power* of the test for each of the four windows (see Methods section) resulted always smaller than 0.01 and greater than 0.99, respectively (Table 2).

**Table 2:** Gene scoring system cross-validation.

Window Width	Mean Value	P-value	Power
100	5.845	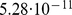	1
75	5.313	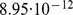	1
50	4.502	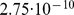	0.999
8	1.151	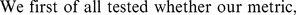	1

Window width indicates the number of top-ranked genes considered in the cross-validation procedure; mean value is the number of disease genes for each window averaged on the 10000 cross-validations; *p-value* and *power* are computed as described in the text.

### Analysis of the top-ranked candidate genes

The candidates ranked according to 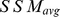 were then examined looking at their functions and expression sites. The twenty top-scored genes are reported in Table 3 together with a brief description of their functions. The number of 20 was arbitrarily chosen, mainly thinking about the intrinsic technical limitations of experimentally testing a great number of genes for disease association – this is actually the reason why such prioritization studies are becoming routine.

**Table 3:** Top-ranked candidate genes.

Gene symbol	Gene description	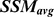	Ear expression	Gene Functions for NSHL
WDR1	WD repeat domain 1	0.55	H. sapiens (ear)^a^	regulation of hair
			M. musculus (inner ear)^a^	cell actin dynamics^f^
ALMS1	Alström syndrome 1	0.53	M. musculus (inner ear)^a^	normal function of cilia [93]
CD151	CD151 molecule	0.52	possible human inner ear component [94]	inner ear ECM assembly [94]
	(Raph blood group)		M. musculus (inner ear)^a^	
CLRN1	clarin 1	0.52	M. musculus (inner ear)^a^	inner ear development^f^
			widely expressed in human^b^	F actin organization^f^
				protein trafficking^f^
ABHD5	abhydrolase domain containing 5	0.52	M. musculus (inner ear)^a^	TG accumulation^f^
				lipid homeostasis^f^
USH1G	Usher syndrome 1G	0.52	H. sapiens (inner ear)^b^^,^ c	cohesion of hair cell bundles^f^
				(ankyrin and pdz domains)
ATP6V0A4	ATPase H 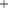 transporting	0.51	H. sapiens (cochlea)^b^ [95]	cochlear pH homeostasis [96]
	lysosomal V0 subunit a4			
PRCD	progressive rod-cone degeneration	0.50	no data	no evidence
KCNQ1	potassium voltage-gated channel	0.50	M. musculus (inner ear)^a^	K 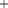 cycling^f^
	KQT-like subfamily member 1			
NUMB	numb homolog (Drosophila)	0.50	H. sapiens (ear)^a^	cell fate determination
			M. musculus (inner ear)^a^	during development^f^
ZAR1	zygote arrest 1	0.50	M. musculus (cochlea, stria vascularis)^g^	no evidence
PTPLA	protein tyrosine phosphatase-like	0.50	H. sapiens (fetal cochlea)^d^	signal transduction^f^
	(proline instead of catalytic arginine)			
	member A			
FLII	flightless I homolog (Drosophila)	0.50	H. sapiens (fetal cochlea)^d^	actin remodeling
PTPN11	protein tyrosine phosphatase	0.49	H. sapiens (ear)^a^	signal transduction^f^
	non-receptor type 11		M. musculus (inner ear)^a^	
TBL1X	transducin (beta)-like 1X-linked	0.49	H. sapiens (fetal cochlea)^d^	signal transduction^f^
			M. musculus (inner ear)	vescicular trafficking^f^
				cytoskeleton assembly^f^
KCNE1L	KCNE1-like	0.49	M. musculus (inner ear)^a^	K 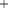 cycling^f^
TIMM8A	translocase of inner mitochondrial	0.49	no data	signal transduction^f^
	membrane 8 homolog A (yeast)			protein transport^f^
ROM1	retinal outer segment	0.49	H. sapiens (fetal cochlea)^d^	cell adhesion^f^
	membrane protein 1			
CC2D2A	coiled-coil and C2	0.49	no data	Ca 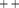 binding^f^
	domain containing 2A			cilia formation^f^
BARHL1	BarH-like homeobox 1	0.48	M.musculus (inner ear)^e^	external sensory organ
				fate determination [97]

Gene expression information are taken from

a NCBI Unigene [98],

b UniProtKB [99],

c HPRD database [100],

d Morton Cochlear EST database [101], NCBI GEO [102],

e the table of gene expression in the developing ear from the Institute of Hearing Research [103],

g Bgee dataBase for Gene Expression Evolution [104] and literature. Gene function information have been inferred from

f NCBI Gene [39] and literature.

Half of them are reported in literature to be expressed in human inner ear or cochlea, despite the very limited availability of gene expression data for these tissues due to the technical difficulties of obtaining undamaged hair-cell samples for gene expression experiments. For the remaining genes, six are reported to be expressed in other organisms' inner ear or cochlea, mainly mouse or chicken, while four have no gene expression data for these tissues. Taken altogether, these are important indications supporting the goodness of the ranking we produced in respect to the NSHL, especially if we think that the initial candidate gene list was not *a priori* filtered by any criterium except that of being all annotated genes located in the susceptibility loci.

Moreover, looking at their functions, we found that most of the top-ranked genes play roles compatible with a possible involvement in NSHL phenotype. Among the most relevant, we identified a) processes of remodeling and organization of actin (WDR1, CLRN1, FLII), an essential component of the hair-cell bundle; b) formation and maintenance of cilia (ALMS1, USH1G, CC2D2A), the sensory organelles devoted to receive the mechanical stimulus; c) 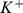 cycling and pH homeostasis in cochlear fluids (ATP6V0A4, KCNQ1, KCNE1L), essentials for the generation and maintenance of the endocochlear potential; d) signal transduction (PTPLA, PTPN11, TBL1X, TIMM8A). They are all important molecular mechanisms underlying the hearing process, which involve the hair cell capability to transduce the mechanical stimulus into electrical signal, as well as the endolymph production and maintenance.

Stronger evidences come from some of the top-ranked genes which are already linked to different syndromic forms of deafness: USH1G for instance is known to cause Usher syndrome type 1G [31], associated with sensorineural hearing impairment; for this gene a possible role in the development and maintenance of the stereocilia bundles is reported by Weil et al. [31]: it might in fact function as an anchoring/scaffolding protein in hair cells and could be involved in the functional network formed by USH1C, CDH23 and MYO7A that is required for cohesion of the growing hair bundle, making its role in the hearing impairment process quite easily explainable. Similarly, KCNE1L has been associated by Piccini et al. [32] to AMME syndrome (Alport syndrome - mental retardation - midface hypoplasia - elliptocytosis) whose symptoms include, among others, hearing loss, and analogous situations are reported also for TIMM8A, involved in Mohr-Tranebjaerg syndrome [33] and Jensen syndrome [34], and ALMS1, involved in Alström syndrome [35]. It is noteworthy that the association of some top-ranked genes to syndromic deafness forms does not exclude them from being good NSHL candidates, as clearly demonstrated by USH1C involved both in Usher syndrome type 1C [36], and NSHL [37], [38], depending on which mutations it undergoes.

**Figure 3:**
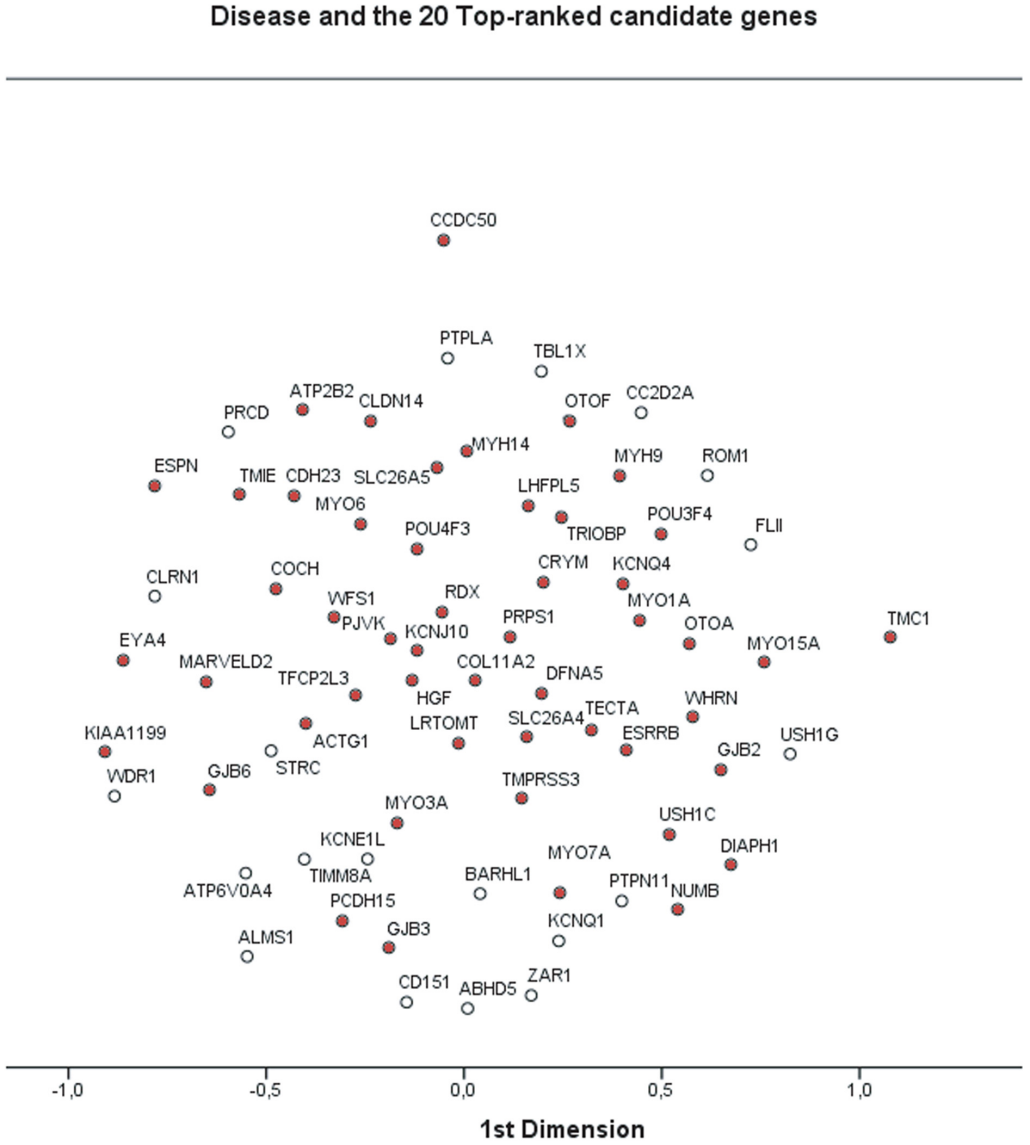
Multidimensional scaling of similarity data to represent the disease and the 20 top-scored candidate genes in a bidimensional space.

Finally, we produced a graphical bidimensional representation of the 20 top-ranked genes together with the disease genes using Proxscal SPSS, which performs multidimensional scaling of similarity data to find a least squares representation of the objects in a low-dimensional space (Figure 3). The proximity of the two gene sets was in this way highlighted; this allowed identifying different groups of NSHL disease genes (red balls in the figure) – namely myosins, connexins, cadherins, ion channels and so forth – and mapping the best candidates within these groups. The inclusion of the top-scored candidate genes did not enlarge the area occupied by the disease genes and their membership to the relative subgroups was mantained in the graphical representation.

Overall, on the basis of these considerations, the majority of them seem to be excellent candidates for subsequent studies on NSHL patients and controls.

### Functional characterization of candidate and disease genes using GO

In order to further investigate the obtained ranking and in order to have a more general picture of the molecular functions, biological processes and cellular components more associated to NSHL, as suggested by both the best candidates and disease genes, we designed and implemented two specific statistical tests that allowed to identify the GO terms more representative of NSHL, exploiting the 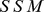 score estimated by our gene scoring system. For the disease genes, we quantified and tested the enrichment of gene-sets defined by functional categories provided by Gene Ontology annotations in disease gene list. In this case the 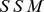 score was used to define the non-disease gene class (see Methods section). For the candidates, we analyzed all GO terms in their annotations, and evaluated the enrichment of the gene set annotated with each of them, by using the 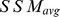 score obtained from our ranking to quantify their association with NSHL (see Materials and Methods section). In this case the 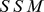 score allowed us, starting from the GO terms associated to all the candidate genes, to identify those GO terms significantly associated with the best candidates, without making any *a priori* decision on which candidates should be considered as the “best” candidates.

This survey had the purpose to examine the ranking on a larger scale – extending the ranking examination to the whole candidate gene set – to possibly suggest non-obvious pathways to further look into when studying NSHL, hence it was devised as a way to look at the results from a different point of view (i.e. moving from a view of NSHL in terms of genes responsible of the disease to a view of NSHL in terms of biological processes, molecular functions and cellular components distinctive of the disease).

We considered as significantly descriptive of the best candidate and disease genes, those GO terms with a *p-value* 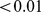 and we ordered them according to their 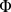 score, function of their *p-value* and specificity in the corpus of the GO annotations.

As for the candidate genes, the enriched terms, divided into biological processes, molecular functions and cellular components (Table 4), include expected concepts such as “auditory receptor cell stereocilium organization” (GO:0060088), “large conductance calcium-activated potassium channel activity” (GO:0060072), “sensory perception of sound” (GO:0007605), “auditory receptor cell stereocilium organization” (GO:0060088), consistent with hearing physiology, as well as less obvious functions or processes such as “regulation of circadian sleep/wake cycle, REM and non-REM sleep” (GO:0042320, GO:0045188), “response to cocaine” (GO:0042220), or “mu-type opioid receptor binding” (GO:0031852), that need further (experimental) investigations. This on the one hand supports again the goodness of the ranking, confirming that the top-scored genes are actually promising candidates for association with NSHL, on the other hand fulfils our initial requirement to suggest new prospective insights in NSHL.

**Table 4:** Enriched biological processes, cellular components and molecular functions for candidate genes.

GO term	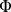 Score	P-value	Definition	Ontology
GO:0060082	17.0	0.002	eye blink reflex	biological process
GO:0014010	16.3	0.005	Schwann cell proliferation	biological process
GO:0034465	16.3	0.002	response to carbon monoxide	biological process
GO:0060231	16.2	0.010	mesenchymal to epithelial transition	biological process
GO:0021771	16.1	0.001	lateral geniculate nucleus development	biological process
GO:0032344	16.0	0.002	regulation of aldosterone metabolic process	biological process
GO:0045759	16.0	0.001	negative regulation of action potential	biological process
GO:0045794	15.9	0.002	negative regulation of cell volume	biological process
GO:0021562	15.7	0.001	vestibulocochlear nerve development	biological process
GO:0050975	15.6	0.005	sensory perception of touch	biological process
GO:0051451	15.6	0.004	myoblast migration	biological process
GO:0031630	15.5	0.005	regulation of synaptic vesicle fusion to presynaptic membrane	biological process
GO:0048790	15.5	0.005	maintenance of presynaptic active zone structure	biological process
GO:0046007	15.2	0.005	negative regulation of activated T cell proliferation	biological process
GO:0046541	15.0	0.005	saliva secretion	biological process
GO:0048676	14.9	0.005	retinal bipolar neuron differentiation	biological process
GO:0045188	14.9	0.001	regulation of circadian sleep/wake cycle, non-REM sleep	biological process
GO:0050916	14.8	0.010	sensory perception of sweet taste	biological process
GO:0035022	14.8	0.009	positive regulation of Rac protein signal transduction	biological process
GO:0042524	14.9	0.005	negative regulation of tyrosine phosphorylation of Stat5 protein	biological process
GO:0060083	14.7	0.002	smooth muscle contraction involved in micturition	biological process
GO:0042320	14.7	0.001	regulation of circadian sleep/wake cycle, REM sleep	biological process
GO:0051496	14.6	0.005	positive regulation of stress fiber formation	biological process
GO:0030007	14.5	0.002	cellular potassium ion homeostasis	biological process
GO:0001661	14.5	0.001	conditioned taste aversion	biological process
GO:0051602	14.4	0.005	response to electrical stimulus	biological process
GO:0032287	14.4	0.004	myelin maintenance in the peripheral nervous system	biological process
GO:0050957	14.2	0.001	equilibrioception	biological process
GO:0045475	14.1	0.002	locomotor rhythm	biological process
GO:0001895	14.1	0.005	retina homeostasis	biological process
GO:0060087	14.0	0.003	relaxation of vascular smooth muscle	biological process
GO:0048484	14.0	0.007	enteric nervous system development	biological process
GO:0022408	14.0	0.005	negative regulation of cell-cell adhesion	biological process
GO:0060088	13.9	0.004	auditory receptor cell stereocilium organization	biological process
GO:0021952	13.8	0.005	central nervous system projection neuron axonogenesis	biological process
GO:0033081	13.8	0.004	regulation of T cell differentiation in the thymus	biological process
GO:0051963	13.0	0.001	regulation of synaptogenesis	biological process
GO:0042220	12.9	0.001	response to cocaine	biological process
GO:0002262	12.9	0.004	myeloid cell homeostasis	biological process
GO:0007019	12.7	0.005	microtubule depolymerization	biological process
GO:0060113	12.5	0.001	inner ear receptor cell differentiation	biological process
GO:0046620	12.4	0.004	regulation of organ growth	biological process
GO:0007605	11.7	0.004	sensory perception of sound	biological process
GO:0045039	11.5	0.005	protein import into mitochondrial inner membrane	biological process
GO:0031667	9.9	0.004	response to nutrient levels	biological process
GO:0019725	7.2	0.004	cellular homeostasis	biological process
GO:0017071	15.9	0.005	intracellular cyclic nucleotide activated cation channel complex	cellular component
GO:0032588	15.7	0.005	trans-Golgi network membrane	cellular component
GO:0032839	14.1	0.004	dendrite cytoplasm	cellular component
GO:0032154	13.9	0.005	cleavage furrow	cellular component
GO:0016011	13.1	0.009	dystroglycan complex	cellular component
GO:0042719	11.6	0.005	mitochondrial intermembrane space protein transporter complex	cellular component
GO:0030660	10.3	0.005	Golgi-associated vesicle membrane	cellular component
GO:0031852	17.0	0.005	mu-type opioid receptor binding	molecular function
GO:0043533	16.3	0.008	inositol 1.3.4.5 tetrakisphosphate binding	molecular function
GO:0060072	15.7	0.002	large conductance calcium-activated potassium channel activity	molecular function
GO:0015266	14.4	0.004	protein channel activity	molecular function
GO:0030346	14.2	0.004	protein phosphatase 2B binding	molecular function
GO:0000822	13.8	0.008	inositol hexakisphosphate binding	molecular function

Candidate gene enriched (*p-value* 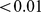) GO terms, sorted according to their 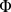 score in each ontology. 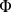 scores take into account the specificity of the terms as described in the text.

As for the disease genes, as expected, the enriched terms are all consistent with the hearing physiology (Table 5). To give some examples, among the most relevant enriched biological processes we found “actin filament-based movement” (GO:0030048), “inner ear morphogenesis” (GO:0042472), “regulation of cell shape” (GO:0008360) and a group involving sensory perception (GO:0007605, GO:0007601, GO:0050957). Likewise, among the enriched cellular components, are “stereocilium” (GO:0032420), “myosin complex” (GO:0016459), “cell junction” (GO:0030054), and among the molecular functions, “actin binding” (GO:0003779), “actin filament binding” (GO:0051015), and so forth.

**Table 5:** Enriched biological processes, cellular components and molecular functions for disease genes.

GO term	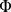 Score	P-value	Definition	Ontology
GO:0007605	150.4	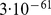	sensory perception of sound	biological process
GO:0007601	22.7	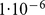	visual perception	biological process
GO:0050957	22.7	0.0001	equilibrioception	biological process
GO:0030048	21.2	0.0001	actin filament-based movement	biological process
GO:0045494	19.5	0.001	photoreceptor cell maintenance	biological process
GO:0050896	18.1	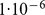	response to stimulus	biological process
GO:0042472	17.8	0.001	inner ear morphogenesis	biological process
GO:0008360	14.2	0.001	regulation of cell shape	biological process
GO:0007155	14.0	0.001	cell adhesion	biological process
GO:0006355	12.0	0.0005	regulation of cellular transcription, DNA-dependent	biological process
GO:0006350	10.2	0.001	cellular transcription	biological process
GO:0006810	7.8	0.009	transport	biological process
GO:0005886	45.5	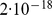	plasma membrane	cellular component
GO:0016021	39.6	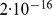	integral to membrane	cellular component
GO:0005737	36.5	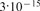	cytoplasm	cellular component
GO:0032420	24.7	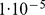	stereocilium	cellular component
GO:0016459	23.0	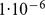	myosin complex	cellular component
GO:0005856	22.9	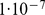	cytoskeleton	cellular component
GO:0030054	22.2	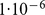	cell junction	cellular component
GO:0031941	21.9	0.0001	filamentous actin	cellular component
GO:0005634	20.1	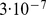	nucleus	cellular component
GO:0016324	19.8	0.0001	apical plasma membrane	cellular component
GO:0001726	18.4	0.001	ruffle	cellular component
GO:0005922	18.1	0.001	connexon complex	cellular component
GO:0005829	16.9	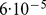	cytosol	cellular component
GO:0005783	16.1	0.0001	endoplasmic reticulum	cellular component
GO:0045202	15.2	0.001	synapse	cellular component
GO:0016020	15.8	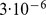	membrane	cellular component
GO:0042995	15.1	0.0001	cell projection	cellular component
GO:0005789	14.4	0.001	endoplasmic reticulum membrane	cellular component
GO:0003779	29.4	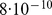	actin binding	molecular function
GO:0005516	28.5	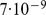	calmodulin binding	molecular function
GO:0051015	20.1	0.0001	actin filament binding	molecular function
GO:0043531	19.8	0.001	ADP binding	molecular function
GO:0003774	18.6	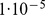	motor activity	molecular function
GO:0005515	16.6	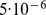	protein binding	molecular function
GO:0004749	16.2	0.001	ribose phosphate diphosphokinase activity	molecular function
GO:0042803	14.2	0.004	protein dimerization activity	molecular function
GO:0005524	12.7	0.0001	ATP binding	molecular function
GO:0043565	12.5	0.001	sequence-specific DNA binding	molecular function
GO:0000166	11.9	0.0001	nucleotide binding	molecular function
GO:0003700	11.6	0.001	transcription factor activity	molecular function

Disease gene enriched (*p-value* 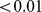) GO terms, sorted according to their 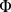 score in each ontology. 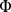 scores take into account the specificity of the terms as described in the text.

Interestingly, among all the enriched terms – for both candidate and disease genes – there is a very small amount of overlapping. Only two biological processes are in fact shared between the two gene lists, “sensory perception of sound” (GO:0007605) and “equilibrioception” (GO:0050957), which are neverthless extremely specific terms – a very small number of gene products are annotated with these terms – both deeply linked to the inner ear function. Looking at the GO graph, however, many of the non-shared terms are interconnected with each other on a larger scale, sharing a common parent at different levels of specificity. This is due to the structure of our algorithm that favours the closeness in the graph of the terms in estimating the similarity between genes. It is noteworthy that with this approach we can think of NSHL from a different perspective, exploring portions of the graph that otherwise would have never been explored.

**Figure 4:**
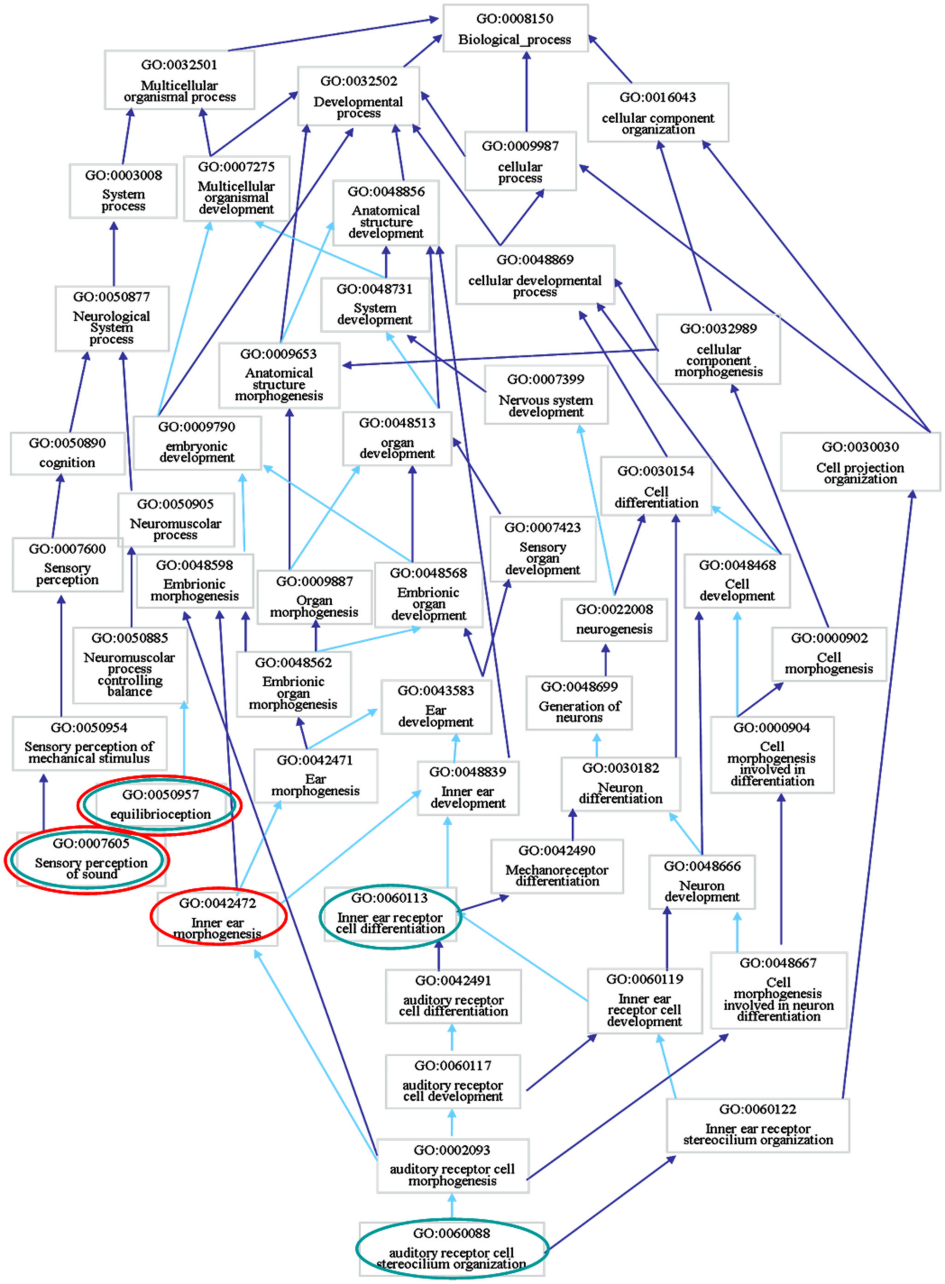
GO subgraph of some disease and candidate gene enriched GO terms. Red circles indicate terms enriched for the disease genes, green circles indicate terms enriched for the candidate genes. Dark blue arrows indicate *is a* relations, light blue arrows indicate *part of* relations between the terms.

In Figure 4 we reported an elucidative example of this issue: by mapping some enriched disease and candidate biological processes to the GO graph, we observed that the addition of “inner ear receptor cell differentiation” (GO:0060113) to the list of NSHL possible biological processes clearly enlarges the NSHL subgraph covering a new branch of the “inner ear development” (GO:0048839) different from the “inner ear morphogenesis” (GO:0042472), while the addition of “auditory receptor cell stereocilium organization” (GO:0060088) narrows and specializes the concept “inner ear morphogenesis” to one of its components.

These findings, as a whole, on the one hand support again the goodness of the ranking, on the other hand they suggest that also some pathways apparently unrelated with NSHL, might deserve future attention by NSHL researchers.

## Discussion

In the perspective of discovering new genes potentially involved in NSHL, we built a gene scoring system integrating Gene Ontology (GO), NCBI Gene and Map Viewer databases, which scores the candidate genes for NSHL by comparing them with the 51 NSHL disease genes already known, relying on the assumption that functionally related genes might contribute to the same (disease) phenotype.

We defined a set of candidate genes for NSHL as all the genes contained in the susceptibility loci known so far, and we prioritized them for the association with the disease, without making any *a priori* selection except that of being annotated with at least one GO term.

We first of all tested whether our metric, 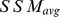, was able to capture the above assumption, verifying that the disease genes are indeed more similar, according to the metric, than two generic human genes. We also demonstrated that our metric is able to pool the disease genes respect to the other human genes, implying that the former are indeed more closely functionally related than the latter: these results therefore justify a prioritization strategy based on the similarity of the candidate genes respect to the disease gene set.

Afterwards, we wanted to investigate to what extent our metric is reliable in ranking candidate genes for their potential role in NSHL manifestation. To this purpose we designed a cross-validation procedure and we obtained excellent results also considering the more disadvantageous condition of ranking eight disease genes in the first 8 positions of a list of more than 8700 genes.

Given these preliminary validations, we are extremely confident that the ranking we produced with respect to NSHL is worthy of attention for future NSHL research plan. Indeed, the top-scored candidate genes play all roles compatible with a possible involvement in NSHL phenotype, representing therefore excellent candidates for subsequent studies on NSHL patients and controls.

However two main limitations of this kind of approach should also be taken into account when looking at these data, both concerning the usage of Gene Ontology annotations to build the gene profiles on which the semantic similarity is measured. One is linked to the current knowledge about the human genome and its content in terms of genes. Indeed, the only prerequisite for a gene to be prioritized by our gene scoring system for a given disease is that of being annotated with at least one GO term, but, as clearly evidenced in this study, we are still far from the complete annotation of the entire human genome, as we were forced to exclude almost half of the possible candidates since they completely lacked GO annotations. This limitation obviously biases the results towards the best studied genes; however it will be progressively overcome in the future, due to the daily updates in this field. The other limitation regards the nature of the associations between GO terms and gene products. All the associations in Gene Ontology fall in five general categories indicating the evidences that support the annotation of a gene to a specific term. Four of these categories comprise exclusively manually-curated associations supported by experimental, computational analysis, author statements or curatorial statements. Unfortunately the great majority of GO associations does not fall in any of these manually-curated categories, being inferred from electronic annotation (IEA), which may open a debate on how reliable and precise they are. At present, given the high percentage of IEA associations in GO, it is not conceivable to discard them and consider only those manually-curated. Other solutions must therefore be devised to address this issue. Future developments of our gene scoring system could for instance take into account this problem by down-weighting the IEA associations respect to those manually-curated. However the quantification of the difference in weight between the manual and electronic associations is not trivial and requires an accurate study of the algorithms behind the electronic associations. We reserve in future to enhance our algorithm in this direction.

Final and essential step to confirm the results presented in this study is however the experimental validation. To this end two main aspects should be taken into account: (i) the accurate study and selection among the top-ranked genes of the most intringuing candidates for NSHL; we think for instance that the first one (WDR1) represents a good starting point, due to both what is known about its functions and structure – it is indeed involved in the organization of the actin, fundamental for the auditory process, and small enough to be quite easily sequenced in a large number of subjects; (ii) an equally accurate selection of the appropriate NSHL patients and controls to be screened for causative mutations; it is advisable for instance to screen these genes on a cohort of patients already excluded to carry mutations in GJB2, due to the high incidence of NSHL cases caused by mutations in this gene, and on a control set appropriately matched for their geographic origin, in order to take into account the geographic distribution of the human DNA sequence variation.

## Methods

A total of 15727 genes (candidate genes) were prioritized for NSHL in this study. We chose as candidate genes all the genes contained in the NSHL susceptibility loci known so far (Tables S1, S2, S3 respectively for NSHL autosomal dominant, autosomal recessive and X-linked, Y-linked and modifier loci), so that all evidences coming from previous linkage analysis studies were taken into account.

We drew the complete disease gene list starting from the Hereditary Hearing Loss Homepage [9] and a team of experts (geneticists and molecular biologists) further analysed the literature to find additional advances in the field by performing multiple queries on PubMed. To the best of our knowledge, 51 genes belong to this category, as reported in Table 1.

For each disease and candidate gene, we extracted all their GO annotations using the file gene2go downloaded on 29th May 2009 from NCBI Entrez Gene ftp site [39]. One out of fifty-one disease genes – MIRN96 – had no GO annotations, therefore it was not included in this study, consequently narrowing the disease gene list to fifty genes. Likewise, 6987 out of 15727 candidate genes had no GO annotations, therefore the candidate gene list was consequently narrowed to 8740 genes.

### Semantic similarity between two genes

As a node-based approach, our metric computes the similarity between two genes by comparing the GO terms describing them, their ancestors, and their descendants in the GO network. It is based on the *Information Content* (*IC*), which gives a measure of how specific and informative a term is. The *IC* of a term 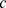 is quantified as the negative log likelihood
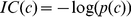
(1)where 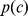 is the probability of occurrence of 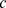 in a specific corpus, which is normally estimated by the frequency of annotation of the term and its children in the GO structure [24], [40].

The concept of *IC* was used by Lin to quantify the semantic similarity between two terms in a tree-structured ontology, measuring the information they share normalized respect to the information contained in their total descriptions. According to Lin's metric [25] the similarity between two terms 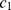 and 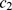 is defined as:
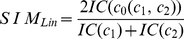
(2)where 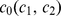 is the most informative common ancestor of the terms 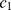 and 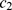, i.e. the common ancestor with the smallest 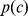.

However, two aspects of this metric limit its application:

- it applies only to trees, where a unique most informative common ancestor between two any given concepts exists;
- it measures the distance between single terms rather than set of terms.

For the first drawback, it is well known that in the case of a direct acyclic graph (DAG), such as GO, two terms can share parents by multiple paths, as multiple parents for each concept are allowed. Therefore, we chose, as 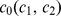, the minimum subsumer between 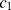 and 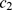 along all their independent paths to the graph root [13].

To address the second issue, we defined our Semantic Similarity Measure (*SSM*) by directly extending Lin's idea to quantify the similarity between two concepts to the comparison between two gene products, i.e. two sets of concepts, therefore measuring the *IC* of the common description of the two gene products, normalized respect to the *IC* of their global description.

Let be
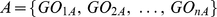


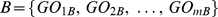
two gene products, annotated respectively with 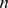 and 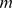 GO terms, that are to be compared. The idea is that each term in 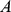 is an independent view of the gene 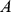 and has to be compared with its counterpart in the 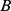 gene annotation, namely the term in 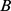 with maximum *IC* for the common description respect to it. In formulas, for the term 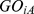, its counterpart 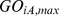 in 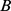 is defined as:
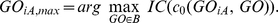
The *IC* for each GO term *c* is estimated using its probability of occurrence 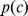 in the corpus of all gene annotations provided by ENGINE database [41], [42]: in details, the probability 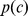 is calculated for every term by counting the number of gene products associated with the term or any of its children, divided by the number of total associations between the GO terms and gene products.

Considering as independent the single views of a gene offered by each of its terms, the semantic similarity of 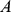 respect to 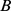 is estimated by the sum of the shared common description *IC*s between each term in 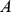 and its counterpart in 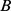 normalized with the *IC* of their global description:
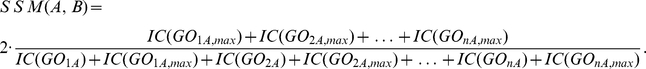



The similarity of the gene 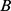 respect to 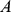 (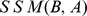) is obtained by inverting the roles of 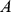 and 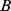 in the above formula. Finally, we defined our Semantic Similarity Measure between 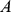 and 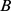, 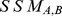, as the mean between the similarity of 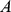 respect to 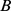 and the similarity of 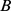 respect to 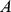:
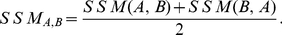
(3)

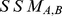
 generates normalized similarity values between 0 and 1: it's equal to 0 for genes annotated with terms that share only the root and equal to 1 for genes annotated with the same terms.

### Validation of the 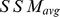 for NSHL gene prioritization

A cross-validation procedure was used to check the reliability of the ranking of candidate genes for their involvement in NSHL. A random set of 8 disease genes was added to the set of candidate genes for 10000 times. Each time the 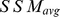 values for this enlarged set of candidates were computed against the remaining disease genes and the number of disease genes 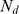 in the first 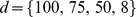 top-ranked positions was counted. The corresponding 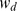 distributions of these countings were then compared with the probabilities of counting 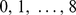 disease genes when a random drawn of 100, 75, 50, 8 genes, respectively, was performed from a set of 8748 genes (8740 candidate genes plus 8 disease genes): in the case of random drawns, the countings are described by a hypergeometric distribution with 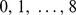 successes for *d* draws without replacement.

More in details, we computed the *p-value* and the *power* of a statistical test on the hypothesis of equal distributions 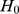 against the hypothesis 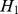 of a greater number of disease genes in the first positions for the 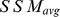 ranked ordering respect to the random ordering. The *p-value* measures the probability to obtain, by random extraction, a number of disease genes 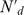 greater than the mean value 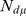 of the number of disease genes found in the 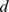 top-ranked positions on the 10000 cross-validations:
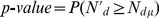
(4)The *p-value* is our estimate of the probability of rejecting 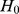 when 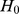 is true: whenever the *p-value* was less than the significance level 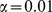, we maintained that the number of disease genes found in the top-ranked positions was statistically significantly greater than that found in random orderings.

The knowledge of the empirical distribution of 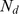 estimated through the cross-validation procedure, allowed us to estimate the *power* 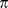 of the test with level 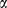: indicating with 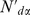 the 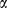-quantile of the hypergeometric distribution, 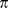 is computed as follows:
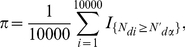
(5)where 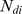 is the number of disease genes found in the first 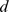 positions for the 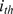 randomization. The larger is the percentage of 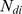 values obtained in cross-validation that are greater than 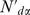, the more effective is the gene prioritization system.

### Functional characterization of candidate and disease genes for NSHL

#### Candidate genes

The statistical test used to identify the most representative GO terms associated with the candidate genes was designed as follows: the null hypothesis is that candidate genes annotated with a particular GO category have an average 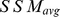 score equal to the average score expected for a random list of candidate genes with the same size, whereas the alternative hypothesis is that the GO category list has a higher average score and, therefore, is supposed to be more associated with the disease than a random candidate gene list. After selecting all the GO terms associated with all the candidate genes, we computed a *p-value* which scores each GO term according to the following strategy: the higher is the number of its associated candidates which obtained in our ranking a high 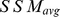 value, the more the GO term is considered enriched in the candidate gene list. This implies that, choosing a significance threshold of 0.01, the GO terms with *p-value* 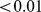 can be considered significantly descriptive of the best candidate genes and consequently significantly associated with the disease. This provides directions for the NSHL researchers about the functions to be more deeply investigated in future laboratory experiments.

The *p-value* for the i-th GO term is computed as follows:
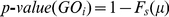
(6)where 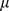 is the average of the 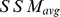 scores resulting for candidate genes annotated with 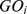, 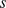 is the number of candidate genes annotated with the 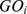 category, 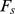 is the empirical cumulative distribution for the 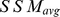 scores, averaged on lists of candidate genes of size 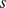. 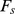 was computed by drawing 10000 random lists of candidate genes of size 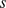 and averaging the respective gene scores.

#### Disease genes

After selecting all the GO terms used to annotate the disease genes, we computed for each GO term a Fisher's exact test *p-value* which scores the GO category (GO Term C) highly for enrichment if many more disease genes than expected belong to the category. The contingency table (Table 6) is built by counting the disease and non-disease genes associated and not associated with the GO category.

**Table 6:** Contingency table.

GO Term C	Disease	Not Disease
Genes annotated with C	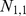	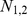
Genes not annotated with C	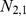	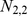

C represents the generic GO term in the disease gene GO annotations.

The definition of the non-disease class is not trivial, as it is not possible to know in advance which candidate genes will be discovered as responsible for NSHL in the future – i.e. it is not possible to discriminate disease and non-disease genes among the candidates. To address this issue we decided to use the distribution of *SSM* scores in the class of candidate genes to define the non-disease class. We considered as non-disease genes the candidate genes with a score less than the 95th percentile of the distribution of candidate gene scores.

The GO terms with a Fisher's test *p-value* smaller than 0.01 are considered significantly over-represented in the list of the disease genes. These provide indications about the main functions and biological processes involved in the hearing mechanisms, taking into account the 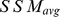 scores computed for our candidate gene list against the NSHL genes at present known.

#### 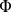 score

For both candidate and disease gene lists their over-represented GO terms are weighted taking into account their specificity in the corpus of the GO annotations as follows:
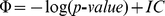
where *IC* is estimated using the probability of occurrence of the GO terms in the corpus of all gene annotations provided by ENGINE database [41], [42].

## Supporting Information

Table S1 NSHL autosomal dominant loci. Locus names and chromosomal locations have been inferred from literature. References are relative to the articles where the locus association to NSHL was identified.
(0.05 MB PDF)

Table S2 NSHL autosomal recessive loci. Locus names and chromosomal locations have been inferred from literature. References are relative to the articles where the locus association to NSHL was identified.
(0.05 MB PDF)

Table S3 NSHL X-linked, Y-linked and modifier loci. Locus names and chromosomal locations have been inferred from literature. References are relative to the articles where the locus association to NSHL was identified.
(0.02 MB PDF)
