# Supplementary material for: Finding New Genes for Non-Syndromic Hearing Loss through an In Silico Prioritization Study
Source: PLoS One. 2010 Sep 28;5(9):e12742. doi: 10.1371/journal.pone.0012742 (PMC2946934; doi:10.1371/journal.pone.0012742)
Supplement: Table S1 — NSHL autosomal dominant loci. Locus names and chromosomal locations have been inferred from literature. References are relative to the articles where the locus association to NSHL was identified. (0.05 MB PDF) [file pone.0012742.s001.pdf]

Table S1. NSHL autosomal dominant loci

LocusName	Position	References
DFNA1	5q31	[1], [2]
DFNA2	1p34	[3, 4]
DFNA3A	13q11	[5, 6]
DFNA3B	13q12	[7]
DFNA4	19q13	[8, 9]
DFNA5	7p15	[10, 11]
DFNA6	4p16	[12–14]
DFNA7	1q21-q23	[15]
DFNA8	11q22-24	[16, 17]
DFNA9	14q12-q13	[18, 19]
DFNA10	6q22-q23	[20–22]
DFNA11	11q13.5	[23–25]
DFNA12	11q22-q24	[16, 17]
DFNA13	6p21	[26, 27]
DFNA14	4p16	[12–14]
DFNA15	5q31	[28]
DFNA16	2q23-q24.3	[29]
DFNA17	22q12.2-q13.3	[30]
DFNA18	3q22	[31]
DFNA19	10 (pericentr.)	[32]
DFNA20	17q25	[33–35]
DFNA21	6p21	[36]
DFNA22	6q13	[37]
DFNA23	14q23	[38]
DFNA24	4q35-qter	[39]
DFNA25	12q22-q24	[40]
DFNA26	17q25	[34, 35]
DFNA27	4q12-q13.1	[41]
DFNA28	8q22	[42]
DFNA30	15q25-26	[43]
DFNA31	6p21.3	[44]
DFNA32	11p15	[45]
DFNA34	1q44	[46]
DFNA36	9q13-q21	[47]
DFNA37	1p21	[48]
DFNA38	4p16	[12–14]
DFNA39	4q21-q22	[49]
DFNA40	16p13.11-p12.2	[50]
DFNA41	12q24-qter	[50]
DFNA42	5q31.1-q32	[51]
DFNA43	2p12	[52]
DFNA44	3q28	[53]
DFNA47	9p21-22	[54]
DFNA48	12q13-q14	[55]
DFNA49	1q21-q23	[56]
DFNA50	7q32	[57]
DFNA51	9q21	[58]
DFNA52	4q28	[51]
DFNA53	14q11-q12	[59]
DFNA54	5q31	[60]
DFNA57	19p13.2	[61]
DFNA59	11p14.2-q12.3	[62]

References

1. Leon P, Raventos H, Lynch E, Morrow J, King M (1992) The gene for an inherited form of deafness maps to chromosome 5q31. Proc Natl Acad Sci U S A 89: 5181-5184.

2. Lynch E, Lee M, Morrow J, Welcsh P, Len P, et al. (1997) Nonsyndromic deafness DFNA1 associated with mutation of a human homolog of the Drosophila gene diaphanous. Science 278: 1315-1318.

3. Coucke P, Van Camp G, Djoyodiharjo B, Smith S, Frants R, et al. (1994) Linkage of autosomal dominant hearing loss to the short arm of chromosome 1 in two families. N Engl J Med 331: 425-431.

4. Kubisch C, Schroeder B, Friedrich T, Ltjohann B, El-Amraoui A, et al. (1999) KCNQ4, a novel potassium channel expressed in sensory outer hair cells, is mutated in dominant deafness. Cell 96: 437-446.

5. Chaïb H, Lina-Granade G, Guilford P, Plauchu H, Levilliers J, et al. (1994) A gene responsible for a dominant form of neurosensory non-syndromic deafness maps to the NSRD1 recessive deafness gene interval. Hum Mol Genet 3: 2219-2222.

6. Denoyelle F, Lina-Granade G, Plauchu H, Bruzzone R, Chab H, et al. (1998) Connexin 26 gene linked to a dominant deafness. Nature 393: 319-320.

7. Grifa A, Wagner C, D'Ambrosio L, Melchionda S, Bernardi F, et al. (1999) Mutations in GJB6 cause nonsyndromic autosomal dominant deafness at DFNA3 locus. Nat Genet 23: 16-18.

8. Chen A, Ni L, Fukushima K, Marietta J, O'Neill M, et al. (1995) Linkage of a gene for dominant non-syndromic deafness to chromosome 19. Hum Mol Genet 4: 1073-1076.

9. Donaudy F, Snoeckx R, Pfister M, Zenner H, Blin N, et al. (2004) Nonmuscle myosin heavy-chain gene MYH14 is expressed in cochlea and mutated in patients affected by autosomal dominant hearing impairment (DFNA4). Am J Hum Genet 74: 770-776.

10. van Camp G, Coucke P, Balemans W, van Velzen D, van de Bilt C, et al. (1995) Localization of a gene for non-syndromic hearing loss (DFNA5) to chromosome 7p15. Hum Mol Genet 4: 2159-2163.

11. Van Laer L, Huizing E, Verstreken M, van Zuijlen D, Wauters J, et al. (1998) Nonsyndromic hearing impairment is associated with a mutation in DFNA5. Nat Genet 20: 194-197.

12. Lesperance M, Hall Jr, Bess F, Fukushima K, Jain P, et al. (1995) A gene for autosomal dominant nonsyndromic hereditary hearing impairment maps to 4p16.3. Hum Mol Genet 4: 1967-1972.

13. Bespalova I, Van Camp G, Bom S, Brown D, Cryns K, et al. (2001) Mutations in the Wolfram syndrome 1 gene (WFS1) are a common cause of low frequency sensorineural hearing loss. Hum Mol Genet 10: 2501-2508.

14. Young T, Ives E, Lynch E, Person R, Snook S, et al. (2001) Non-syndromic progressive hearing loss DFNA38 is caused by heterozygous missense mutation in the Wolfram syndrome gene WFS1. Hum Mol Genet 10: 2509-2514.

15. Fagerheim T, Nilssen O, Raeymaekers P, Brox V, Moum T, et al. (1996) Identification of a new locus for autosomal dominant non-syndromic hearing impairment (DFNA7) in a large Norwegian family. Hum Mol Genet 5: 1187-1191.

16. Verhoeven K, Van Camp G, Govaerts P, Balemans W, Schatteman I, et al. (1997) A gene for autosomal dominant nonsyndromic hearing loss (DFNA12) maps to chromosome 11q22-24. Am J Hum Genet 60: 1168-1173.

17. Verhoeven K, Van Laer L, Kirschhofer K, Legan P, Hughes D, et al. (1998) Mutations in the human alpha-tectorin gene cause autosomal dominant non-syndromic hearing impairment. Nat Genet 19: 60-62.

18. Manolis E, Yandavi N, Nadol JJ, Eavey R, McKenna M, et al. (1996) A gene for non-syndromic autosomal dominant progressive postlingual sensorineural hearing loss maps to chromosome 14q12-13. Hum Mol Genet 5: 1047-1050.

19. Robertson N, Lu L, Heller S, Merchant S, Eavey R, et al. (1998) Mutations in a novel cochlear gene cause DFNA9, a human nonsyndromic deafness with vestibular dysfunction. Nat Genet 20: 299-303.

20. O'Neill M, Marietta J, Nishimura D, Wayne S, G VC, et al. (1996) A gene for autosomal dominant late-onset progressive non-syndromic hearing loss, DFNA10, maps to chromosome 6. Hum Mol Genet 5: 853-856.

21. Wayne S, Robertson N, DeClau F, Chen N, Verhoeven K, et al. (2001) Mutations in the transcriptional activator EYA4 cause late-onset deafness at the DFNA10 locus. Hum Mol Genet 10: 195-200.

22. Verhoeven K, Fagerheim T, Prasad S, Wayne S, De Clau F, et al. (2000) Refined localization and two additional linked families for the DFNA10 locus for nonsyndromic hearing impairment. Hum Genet 107: 7-11.

23. Guilford P, Ayadi H, Blanchard S, Chaib H, Le Paslier D, et al. (1994) A human gene responsible for neurosensory, non-syndromic recessive deafness is a candidate homologue of the mouse sh-1 gene. Hum Mol Genet 3: 989-993.

24. Liu X, Walsh J, Mburu P, Kendrick-Jones J, Cope M, et al. (1997) Mutations in the myosin VIIA gene cause non-syndromic recessive deafness. Nat Genet 16: 188-190.

25. Weil D, Kssel P, Blanchard S, Lvy G, Levi-Acobas F, et al. (1997) The autosomal recessive isolated deafness, DFNB2, and the Usher 1B syndrome are allelic defects of the myosin-VIIA gene. Nat Genet 16: 191-193.

26. Brown M, Tomek M, Van Laer L, Smith S, Kenyon J, et al. (1997) A novel locus for autosomal dominant nonsyndromic hearing loss, DFNA13, maps to chromosome 6p. Am J Hum Genet 61: 924-927.

27. McGuirt W, Prasad S, Griffith A, Kunst H, Green G, et al. (1999) Mutations in COL11A2 cause non-syndromic hearing loss (DFNA13). Nat Genet 23: 413-419.

28. Vahava O, Morell R, Lynch E, Weiss S, Kagan M, et al. (1998) Mutation in transcription factor POU4F3 associated with inherited progressive hearing loss in humans. Science 279: 1950-1954.

29. Fukushima K, Kasai N, Ueki Y, Nishizaki K, Sugata K, et al. (1999) A gene for fluctuating, progressive autosomal dominant nonsyndromic hearing loss, DFNA16, maps to chromosome 2q23-24.3. Am J Hum Genet 65: 141-150.

30. Lalwani A, Luxford W, Mhatre A, Attaie A, Wilcox E, et al. (1999) A new locus for nonsyndromic hereditary hearing impairment, DFNA17, maps to chromosome 22 and represents a gene for cochleosaccular degeneration. Am J Hum Genet 64: 318-323.

31. Bonsch D, Scheer P, Neumann C, Lang-Roth R, Seifert E, et al. (2001) A novel locus for autosomal dominant, non-syndromic hearing impairment (DFNA18) maps to chromosome 3q22 immediately adjacent to the DM2 locus. Europ J Hum Genet 9: 165-170.

32. Green, et al (1998) Abstract 107. In: The Molecular Biology of Hearing and Deafness Meeting. Bethesda, Maryland.

33. Morell R, Friderici K, Wei S, Elfenbein J, Friedman T, et al. (2000) A new locus for late-onset, progressive, hereditary hearing loss DFNA20 maps to 17q25. Genomics 63: 1-6.

34. Zhu M, Yang T, Wei S, DeWan A, Morell R, et al. (2003) Mutations in the gamma-actin gene (ACTG1) are associated with dominant progressive deafness (DFNA20/26). Am J Hum Genet 73: 1082-1091.

35. van Wijk E, Krieger E, Kemperman M, De Leenheer E, Huygen P, et al. (2003) A mutation in the gamma actin 1 (ACTG1) gene causes autosomal dominant hearing loss (DFNA20/26). J Med Genet 40: 879-884.

36. Kunst H, Marres H, Huygen P, Van Duijnhoven G, Krebsova A, et al. (20009) Non-syndromic autosomal dominant progressive non-specific mid-frequency sensorineural hearing impairment with childhood to late adolescence onset (DFNA21). Clin Otolaryng 25: 45-54.

37. Melchionda S, Ahituv N, Bisceglia L, Sobe T, Glaser F, et al. (2001) MYO6, the human homologue of the gene responsible for deafness in Snell's waltzer mice, is mutated in autosomal dominant nonsyndromic hearing loss. Am J Hum Genet 69: 635-640.

38. Ruf R, Xu P, Silvius D, Otto E, Beekmann F, et al. (2004) SIX1 mutations cause branchio-oto-renal syndrome by disruption of EYA1-SIX1-DNA complexes. Proc Natl Acad Sci U S A 101: 8090-8095.

39. Häfner F, Salam A, Linder T, Balmer D, Baumer A, et al. (2000) A novel locus (DFNA24) for prelingual nonprogressive autosomal dominant nonsyndromic hearing loss maps to 4q35-qter in a large Swiss German kindred. Am J Hum Genet 66: 1437-1442.

40. Petek E, Windpassinger C, Mach M, Rauter L, Scherer S, et al. (2003) Molecular characterization of a 12q22-q24 deletion associated with congenital deafness: confirmation and refinement of the DFNA25 locus. Am J Med Genet A 117: 122-126.

41. Peters L, Fridell R, Boger E, San Agustin T, Madeo A, et al. (2008) A locus for autosomal dominant progressive non-syndromic hearing loss, DFNA27, is on chromosome 4q12-13.1. Clin Genet 73: 367-372.

42. Peters L, Anderson D, Griffith A, Grundfast K, San Agustin T, et al. (2002) Mutation of a transcription factor, TFCP2L3, causes progressive autosomal dominant hearing loss, DFNA28. Hum Mol Genet 11: 2877-2885.

43. Mangino M, Flex E, Capon F, Sangiuolo F, Carraro E, et al. (2001) Mapping of a new autosomal dominant nonsyndromic hearing loss locus (DFNA30) to chromosome 15q25-26. Eur J Hum Genet 9: 667-671.

44. Snoeckx R, Kremer H, Ensink R, Flothmann K, de Brouwer A, et al. (2004) A novel locus for autosomal dominant non-syndromic hearing loss, DFNA31, maps to chromosome 6p21.3. J Med Genet 41: 11-13.

45. Li X, Saal H, Friedman T, Friedman R (2000) A new gene for autosomal dominant nonsyndromic sensorineural hearing loss (DFNA32) maps to 11p15. ASHG Annual Meeting Abstract: 1727.

46. Kurima K, Szymko Y, Rudy S, Morell RJ, Friedman TB, et al. (2000) Genetic map localization of DFNA34 and DFNA26, two autosomal dominant non-syndromic deafness loci. ASHG Annual Meeting Abstract: 1654.

47. Kurima K, Peters L, Yang Y, Riazuddin S, Ahmed Z, et al. (2002) Dominant and recessive deafness caused by mutations of a novel gene, TMC1, required for cochlear hair-cell function. Nat Genet 30: 277-284.

48. Talebizadeh Z, Kenyon J, Askew J, Smith S (2000) A new locus for dominant progressive hearing loss DFN37 mapped to chromosome 1p21. ASHG Annual Meeting Abstract: 1740.

49. Xiao S, Yu C, Chou X, Yuan W, Wang Y, et al. (2001) Dentinogenesis imperfecta 1 with or without progressive hearing loss is associated with distinct mutations in DSPP. Nature Genet 27: 201-204.

50. Chen H, Phillips H, Callen D, Kim R, Wistow G, et al. (1992) Localization of the human gene for mu-crystallin to chromosome 16p. Genomics 14: 1115-1116.

51. Xia J, Deng H, Feng Y, Zhang H, Pan Q, et al. (2002) A novel locus for autosomal dominant nonsyndromic hearing loss identified at 5q31.1-32 in a Chinese pedigree. J Hum Genet 47: 635-640.

52. Flex E, Mangino M, Mazzoli M, Martini A, Migliosi V, et al. (2003) Mapping of a new autosomal dominant non-syndromic hearing loss locus (DFNA43) to chromosome 2p12. J Med Genet 40: 278-281.

53. Modamio-Høybjør S, Moreno-Pelayo M, Menca A, del Castillo I, Chardenoux S, et al. (2003) A novel locus for autosomal dominant nonsyndromic hearing loss (DFNA44) maps to chromosome 3q28-29. Hum Genet 112: 24-28.

54. D'Adamo P, Donaudy F, D'Eustacchio A, Di Iorio E, Melchionda S, et al. (2003) A new locus (DFNA47) for autosomal dominant non-syndromic inherited hearing loss maps to 9p21-22 in a large Italian family. Eur J Hum Genet 11: 121-124.

55. D'Adamo P, Pinna M, Capobianco S, Cesarani A, D'Eustacchio A, et al. (2003) A novel autosomal dominant non-syndromic deafness locus (DFNA48) maps to 12q13-q14 in a large Italian family. Hum Genet 112: 319-320.

56. Moreno-Pelayo M, Modamio-Høybjør S, Menca A, del Castillo I, Chardenoux S, et al. (2003) DFNA49, a novel locus for autosomal dominant non-syndromic hearing loss, maps proximal to DFNA7/DFNM1 region on chromosome 1q21-q23. J Med Genet 40: 832-836.

57. Modamio-Høybjør S, Moreno-Pelayo M, Menca A, del Castillo I, Chardenoux S, et al. (2004) A novel locus for autosomal dominant nonsyndromic hearing loss, DFNA50, maps to chromosome 7q32 between the DFNB17 and DFNB13 deafness loci. J Med Genet 41: e14.

58. Hereditary Hearing Loss Homepage. URL `http://webh01.ua.ac.be/hhh/`.

59. Yan D, Ke X, Blanton S, Ouyang X, Pandya A, et al. (2006) A novel locus for autosomal dominant non-syndromic deafness, DFNA53, maps to chromosome 14q11.2-q12. J Med Genet 43: 170-174.

60. Gürtler N, Kim Y, Mhatre A, Schlegel C, Mathis A, et al. (2004) DFNA54, a third locus for low-frequency hearing loss. J Mol Med 82: 775-780.

61. Bönsch D, Schmidt C, Scheer P, Bohlender J, Neumann C, et al. (2008) A new locus for an autosomal dominant, non-syndromic hearing impairment (DFNA57) located on chromosome 19p13.2 and overlapping with DFNB15. HNO 56: 177-182.

62. Chatterjee A, Jalvi R, Pandey N, Rangasayee R, Anand A (2009) A novel locus DFNA59 for autosomal dominant nonsyndromic hearing loss maps at chromosome 11p14.2-q12.3. Hum Genet 124: 669-675.
