# Supplementary material for: Finding New Genes for Non-Syndromic Hearing Loss through an In Silico Prioritization Study
Source: PLoS One. 2010 Sep 28;5(9):e12742. doi: 10.1371/journal.pone.0012742 (PMC2946934; doi:10.1371/journal.pone.0012742)
Supplement: Table S2 — NSHL autosomal recessive loci. Locus names and chromosomal locations have been inferred from literature. References are relative to the articles where the locus association to NSHL was identified. (0.05 MB PDF) [file pone.0012742.s002.pdf]

Table S2. NSHL autosomal recessive loci

LocusName	Position	References
DFNB1	13q11-q12	[1–3]
DFNB2	11q13.5	[4–6]
DFNB3	17p11.2	[7]
DFNB4	7q31	[8]
DFNB5	14q12	[9, 10]
DFNB6	3p21	[11]
DFNB7	9q13-q21	[12]
DFNB8	21q22	[13]
DFNB9	2p22-p23	[14]
DFNB10	21q22.3	[15, 16]
DFNB11	9q13-q21	[17]
DFNB12	10q21-q22	[14]
DFNB13	7q34-36	[18]
DFNB14	7q31	[19]
DFNB15	3q21-q25; 19p13	[20]
DFNB16	15q15	[21, 22]
DFNB17	7q31	[23]
DFNB18	11p14-15.1	[24]
DFNB19	18p11	[25]
DFNB20	11q25-qter	[26]
DFNB21	11q22-q24	[27]
DFNB22	16p12.2	[28]
DFNB23	10q21	[29]
DFNB24	11q23	[30]
DFNB25	4p15.3-q12	[31]
DFNB26	4q31	[32]
DFNB27	2q23-q31	[33]
DFNB28	22q13	[34]
DFNB29	21q22	[35]
DFNB30	10p11.1	[36]
DFNB31	q32-q34	[37]
DFNB32	1p13.3-p22.1	[38]
DFNB33	10p11.23-q21.1	[39]
DFNB35	14q24.1-q24.3	[40]
DFNB36	1p36.3-p36.1	[41]
DFNB37	6q13	[42]
DFNB38	6q26-q27	[43]
DFNB39	7q11.22-q21.12	[44]
DFNB40	22q11.21-q12.1	[45]
DFNB42	3q13.31-q22.3	[46]
DFNB44	7p14.1-q11.22	[47]
DFNB45	1q43-q44	[48]
DFNB46	18p11.32-p11.31	[49]
DFNB47	2p25.1-p24.3	[50]
DFNB48	15q23-q25.1	[51]
DFNB49	5q13.1	[52, 53]
DFNB50	12q23	[31]
DFNB51	11p13-p12	[54]
DFNB53	6p21.3	[55]
DFNB55	4q12-q13.2	[56]
DFNB57	10q23.1-q26.11	[31]
DFNB58	2q14.1-q21.2	[31]
DFNB59	2q31.1-q31.3	[57]
DFNB60	5q22-q31	[31]
DFNB61	7q22.1	[31]
DFNB62	12p13.2-p11.23	[58]
DFNB63	11q13.3-q13.4	[59, 60]
DFNB65	20q13.2-q13.32	[61]
DFNB66	6p21.2-p22.3	[62]
DFNB67	6p21.1-p22.3	[63]
DFNB68	19p13.2	[64]
DFNB72	19p13.3	[65]

References

1. Guilford P, Ben Arab S, Blanchard S, Levilliers J, Weissenbach J, et al. (1994) A non-syndrome form of neurosensory, recessive deafness maps to the pericentromeric region of chromosome 13q. Nat Genet 6: 24-28.

2. Brown K, Janjua A, Karbani G, Parry G, Noble A, et al. (1996) Linkage studies of non-syndromic recessive deafness (NSRD) in a family originating from the Mirpur region of Pakistan maps DFNB1 centromeric to D13s175. Hum Mol Genet 5: 169-173.

3. Kelsell D, Dunlop J, Stevens H, Lench N, Liang J, et al. (1997) Connexin 26 mutations in hereditary non-syndromic sensorineural deafness. Nature 387: 80-83.

4. Guilford P, Ayadi H, Blanchard S, Chaib H, Le Paslier D, et al. (1994) A human gene responsible for neurosensory, non-syndromic recessive deafness is a candidate homologue of the mouse sh-1 gene. Hum Mol Genet 3: 989-993.

5. Liu X, Walsh J, Mburu P, Kendrick-Jones J, Cope M, et al. (1997) Mutations in the myosin VIIA gene cause non-syndromic recessive deafness. Nat Genet 16: 188-190.

6. Weil D, Kssel P, Blanchard S, Lvy G, Levi-Acobas F, et al. (1997) The autosomal recessive isolated deafness, DFNB2, and the Usher 1B syndrome are allelic defects of the myosin-VIIA gene. Nat Genet 16: 191-193.

7. Liang Y, Wang A, Probst F, Arhya I, Barber T, et al. (1998) Genetic mapping refines DFNB3 to 17p11.2, suggests multiple alleles of DFNB3, and supports homology to the mouse model shaker-2. Am J Hum Genet 62: 904-915.

8. Baldwin C, Weiss S, Farrer L, De Stefano A, Adair R, et al. (1995) Linkage of congenital, recessive deafness (DFNB4) to chromosome 7q31 and evidence for genetic heterogeneity in the Middle Eastern Druze population. Hum Mol Genet 4: 1637-1642.

9. Fukushima K, Ramesh A, Srisailapathy C, Ni L, Chen A, et al. (1995) Consanguineous nuclear families used to identify a new locus for recessive non-syndromic hearing loss on 14q. Am J Hum Genet 4: 1643-1648.

10. Petit C (1996) Genes responsible for human hereditary deafness: symphony of a thousand. Nat Genet 14: 385-391.

11. Fukushima K, Ramesh A, Srisailapathy C, Ni L, Wayne S, et al. (1995) An autosomal recessive nonsyndromic form of sensorineural hearing loss maps to 3p-DFNB6. Genome Res 5: 305-308.

12. Jain P, Fukushima K, Deshmukh D, Ramesh A, Thomas E, et al. (1995) A human recessive neurosensory nonsyndromic hearing impairment locus is potential homologue of murine deafness (dn) locus. Hum Mol Genet 4: 2391-2394.

13. Veske A, Oehlmann R, Younus F, Mohyuddin A, Mller-Myhsok B, et al. (1996) Autosomal recessive non-syndromic deafness locus (DFNB8) maps on chromosome 21q22 in a large consanguineous kindred from Pakistan. Hum Mol Genet 5: 165-168.

14. Chaïb H, Lina-Granade G, Guilford P, Plauchu H, Levilliers J, et al. (1994) A gene responsible for a dominant form of neurosensory non-syndromic deafness maps to the NSRD1 recessive deafness gene interval. Hum Mol Genet 3: 2219-2222.

15. Bonné-Tamir B, De Stefano A, Briggs C, Adair R, Franklyn B, et al. (1996) Linkage of congenital recessive deafness (gene DFNB10) to chromosome 21q22.3. Am J Hum Genet 58: 1254-1259.

16. Berry A, Scott H, Kudoh J, Talior I, Korostishevsky M, et al. (2000) Refined localization of autosomal recessive nonsyndromic deafness DFNB10 locus using 34 novel microsatellite markers, genomic structure, and exclusion of six known genes in the region. Genomics 68: 22-29.

17. Scott D, Carmi R, Elbedour K, Yosefsberg S, Stone E, et al. (1996) An autosomal recessive nonsyndromic-hearing-loss locus identified by DNA pooling using two inbred Bedouin kindreds. Am J Hum Genet 59: 385-391.

18. Mustapha M, Chardenoux S, Nieder A, Salem N, Weissenbach J, et al. (1998) A sensorineural progressive autosomal recessive form of isolated deafness, DFNB13, maps to chromosome 7q34-q36. Eur J Hum Genet 6: 245-250.

19. Mustapha M, Salem N, Weil D, el Zir E, Loiselet J, et al. (1998) Identification of a locus on chromosome 7q31, DFNB14, responsible for prelingual sensorineural non-syndromic deafness. Eur J Hum Genet 6: 548-551.

20. Chen A, Wayne S, Bell A, Ramesh A, Srisailapathy C, et al. (1997) New gene for autosomal recessive non-syndromic hearing loss maps to either chromosome 3q or 19p. Am J Hum Genet 7: 467-471.

21. Villamar M, del Castillo I, Valle N, Romero L, Moreno F (1999) Deafness locus DFNB16 is located on chromosome 15q13-q21 within a 5-cM interval flanked by markers D15S994 and D15S132. Am J Hum Genet 64: 1238-1241.

22. Verpy E, Masmoudi S, Zwaenepoel I, Leibovici M, Hutchin T, et al. (2001) Mutations in a new gene encoding a protein of the hair bundle cause non-syndromic deafness at the DFNB16 locus. Nat Genet 29: 345-349.

23. Greinwald JJ, Wayne S, Chen A, Scott D, Zbar R, et al. (1998) Localization of a novel gene for nonsyndromic hearing loss (DFNB17) to chromosome region 7q31. Am J Med Genet 78: 107-113.

24. Jain P, Lalwani A, Li X, Singleton T, Smith T, et al. (1998) A gene for recessive nonsyndromic sensorineural deafness (DFNB18) maps to the chromosomal region 11p14-p15.1 containing the Usher syndrome type 1C gene. Genomics 50: 290-292.

25. Green, et al (1998) Abstract 107. In: The Molecular Biology of Hearing and Deafness Meeting. Bethesda, Maryland.

26. Moynihan L, Houseman M, Newton V, Mueller R, Lench N (1999) DFNB20: a novel locus for autosomal recessive, non-syndromal sensorineural hearing loss maps to chromosome 11q25-qter. Eur J Hum Genet 7: 243-246.

27. Mustapha M, Weil D, Chardenoux S, Elias S, El-Zir E, et al. (1999) An alpha-tectorin gene defect causes a newly identified autosomal recessive form of sensorineural pre-lingual non-syndromic deafness, DFNB21. Hum Mol Genet 8: 409-412.

28. Zwaenepoel I, Mustapha M, Leibovici M, Verpy E, Goodyear R, et al. (2002) Otoancorin, an inner ear protein restricted to the interface between the apical surface of sensory epithelia and their overlying acellular gels, is defective in autosomal recessive deafness DFNB22. Proc Natl Acad Sci U S A 99: 6240-6245.

29. Ahmed Z, Riazuddin S, Ahmad J, Bernstein S, Guo Y, et al. (2003) PCDH15 is expressed in the neurosensory epithelium of the eye and ear and mutant alleles are responsible for both USH1F and DFNB23. Hum Mol Genet 12: 3215-3223.

30. Khan S, Ahmed Z, Shabbir M, Kitajiri S, Kalsoom S, et al. (2007) Mutations of the RDX gene cause nonsyndromic hearing loss at the DFNB24 locus. Hum Mutat 28: 417-423.

31. Hereditary Hearing Loss Homepage. URL `http://webh01.ua.ac.be/hhh/`.

32. Riazuddin S, Castelein C, Ahmed Z, Lalwani A, Mastroianni M, et al. (2000) Dominant modifier DFNM1 suppresses recessive deafness DFNB26. Nat Genet 26: 431-434.

33. Pulleyn L, Jackson A, Roberts E, Carridice A, Muxworthy C, et al. (2000) A new locus for autosomal recessive non-syndromal sensorineural hearing impairment (DFNB27) on chromosome 2q23-q31. Eur J Hum Genet 8: 991-993.

34. Shahin H, Walsh T, Sobe T, Abu Sa'ed J, Abu Rayan A, et al. (2006) Mutations in a novel isoform of TRIOBP that encodes a filamentous-actin binding protein are responsible for DFNB28 recessive nonsyndromic hearing loss. Am J Hum Genet 78: 144-152.

35. Wilcox E, Burton Q, Naz S, Riazuddin S, Smith T, et al. (2001) Mutations in the gene encoding tight junction claudin-14 cause autosomal recessive deafness DFNB29. Cell 104: 165-172.

36. Walsh T, Walsh V, Vreugde S, Hertzano R, Shahin H, et al. (2002) From flies' eyes to our ears: mutations in a human class III myosin cause progressive nonsyndromic hearing loss DFNB30. Proc Natl Acad Sci U S A 99: 7518-7523.

37. Mustapha M, Chouery E, Chardenoux S, Naboulsi M, Paronnaud J, et al. (2002) DFNB31, a recessive form of sensorineural hearing loss, maps to chromosome 9q32-34. Eur J Hum Genet 10: 210-212.

38. Masmoudi S, Tlili A, Majava M, Ghorbel A, Chardenoux S, et al. (2002) Mapping of a new autosomal recessive nonsyndromic hearing loss locus (DFNB32) to chromosome 1p13.3-22.1. Eur J Hum Genet 11: 185-188.

39. Belguith H, Masmoudi S, Medlej-Hashim M, Chouery E, Weil D, et al. (2009) Re-assigning the DFNB33 locus to chromosome 10p11.23-q21.1. Eur J Hum Genet 17: 122-124.

40. Ansar M, Din M, Arshad M, Sohail M, Faiyaz-Ul-Haque M, et al. (2003) A novel autosomal recessive non-syndromic deafness locus (DFNB35) maps to 14q24.1-14q24.3 in large consanguineous kindred from Pakistan. Eur J Hum Genet 11: 77-80.

41. Naz S, Griffith A, Riazuddin S, Hampton L, Battey JJ, et al. (2004) Mutations of ESPN cause autosomal recessive deafness and vestibular dysfunction. J Med Genet 41: 591-595.

42. Ahmed Z, Morell R, Riazuddin S, Gropman A, Shaukat S (2003) Localization of a novel autosomal recessive non-syndromic hearing impairment locus (DFNB38) to 6q26-q27 in a consanguineous kindred from Pakistan. Am J Hum Genet 72: 1315-1322.

43. Ansar M, Ramzan M, Pham T, Yan K, Jamal S, et al. (2003) Localization of a novel autosomal recessive non-syndromic hearing impairment locus (DFNB38) to 6q26-q27 in a consanguineous kindred from Pakistan. Hum Hered 55: 71-74.

44. Wajid M, Abbasi A, Ansar M, Pham T, Yan K, et al. (2003) DFNB39, a recessive form of sensorineural hearing impairment, maps to chromosome 7q11.22-q21.12. Eur J Hum Genet 11: 812-815.

45. Delmaghani S, Aghaie A, Compain-Nouaille S, Ataie A, Lemainque A, et al. (2003) DFNB40, a recessive form of sensorineural hearing loss, maps to chromosome 22q11.21-12.1. Eur J Hum Genet 11: 816-818.

46. Aslam M, Wajid M, Chahrour M, Ansar M, Haque S, et al. (2005) A novel autosomal recessive nonsyndromic hearing impairment locus (DFNB42) maps to chromosome 3q13.31-q22.3. Am J Med Genet A 133: 18-22.

47. Ansar M, Chahrour M, Amin Ud Din M, Arshad M, Haque S, et al. (2004) DFNB44, a novel autosomal recessive non-syndromic hearing impairment locus, maps to chromosome 7p14.1-q11.22. Hum Hered 57: 195-199.

48. Bhatti A, Lee K, McDonald M, Hassan M, Gutala R, et al. (2008) Mapping of a new autosomal recessive non-syndromic hearing impairment locus (DFNB45) to chromosome 1q43-q44. Clin Genet 73: 395-398.

49. Mir A, Ansar M, Chahrour M, Pham T, Wajid M, et al. (2005) Mapping of a novel autosomal recessive nonsyndromic deafness locus (DFNB46) to chromosome 18p11.32-p11.31. Am J Med Genet A 133: 23-26.

50. Hassan M, Santos R, Rafiq M, Chahrour M, Pham T, et al. (2006) A novel autosomal recessive non-syndromic hearing impairment locus (DFNB47) maps to chromosome 2p25.1-p24.3. Hum Genet 118: 605-610.

51. Ahmad J, Khan S, Khan S, Ramzan K, Riazuddin S, et al. (2005) DFNB44, a novel autosomal recessive non-syndromic hearing impairment locus, maps to chromosome 7p14.1-q11.22. Hum Genet 116: 407-412.

52. Ramzan K, Shaikh R, Ahmad J, Khan S, Riazuddin S, et al. (2005) A new locus for nonsyndromic deafness DFNB49 maps to chromosome 5q12.3-q14.1. Hum Genet 116: 17-22.

53. Riazuddin S, Ahmed Z, Fanning A, Lagziel A, Kitajiri S, et al. (2006) Tricellulin is a tight-junction protein necessary for hearing. Am J Hum Genet 79: 1040-1051.

54. Shaikh R, Ramzan K, Nazli S, Sattar S, Khan S, et al. (2005) A new locus for nonsyndromic deafness DFNB51 maps to chromosome 11p13-p12. Am J Med Genet A 138: 392-395.

55. Chen W, Kahrizi K, Meyer N, Riazalhosseini Y, Van Camp G, et al. (2005) Mutation of COL11A2 causes autosomal recessive non-syndromic hearing loss at the DFNB53 locus. J Med Genet 42: 61.

56. Irshad S, Santos R, Muhammad D, Lee K, McArthur N, et al. (2005) Localization of a novel autosomal recessive non-syndromic hearing impairment locus DFNB55 to chromosome 4q12-q13.2. Clin Genet 68: 262-267.

57. Delmaghani S, del Castillo F, Michel V, Leibovici M, Aghaie A, et al. (2006) Mutations in the gene encoding pejvakin, a newly identified protein of the afferent auditory pathway, cause DFNB59 auditory neuropathy. Nat Genet 38: 770-778.

58. Ali G, Santos R, John P, Wambangco M, Lee K, et al. (2006) The mapping of DFNB62, a new locus for autosomal recessive non-syndromic hearing impairment, to chromosome 12p13.2-p11.23. Clin Genet 69: 429-433.

59. Khan S, Riazuddin S, Tariq M, Anwar S, Shabbir M, et al. (2007) Autosomal recessive nonsyndromic deafness locus DFNB63 at chromosome 11q13.2-q13.3. Hum Genet 120: 789-793.

60. Tlili A, Masmoudi S, Dhouib H, Bouaziz S, Rebeh I, et al. (2007) Localization of a novel autosomal recessive non-syndromic hearing impairment locus DFNB63 to chromosome 11q13.3-q13.4. Ann Hum Genet 71: 271-275.

61. Tariq A, Santos R, Khan M, Lee K, Hassan M, et al. (2006) Localization of a novel autosomal recessive nonsyndromic hearing impairment locus DFNB65 to chromosome 20q13.2-q13.32. J Mol Med 84: 484-490.

62. Tlili A, Mnnikk M, Charfedine I, Lahmar I, Benzina Z, et al. (2005) A novel autosomal recessive non-syndromic deafness locus, DFNB66, maps to chromosome 6p21.2-22.3 in a large Tunisian consanguineous family. Hum Hered 60: 123-128.

63. Shabbir M, Ahmed Z, Khan S, Riazuddin S, Waryah A, et al. (2006) Mutations of human TMHS cause recessively inherited non-syndromic hearing loss. J Med Genet 43: 634-640.

64. Santos R, Hassan M, Sikandar S, Lee K, Ali G, et al. (2006) DFNB68, a novel autosomal recessive non-syndromic hearing impairment locus at chromosomal region 19p13.2. Hum Genet 120: 85-92.

65. Ain Q, Nazli S, Riazuddin S, Jaleel A, Riazuddin S, et al. (2007) The autosomal recessive nonsyndromic deafness locus DFNB72 is located on chromosome 19p13.3. Hum Genet 122: 445-450.
