# Supplementary material for: Finding New Genes for Non-Syndromic Hearing Loss through an In Silico Prioritization Study
Source: PLoS One. 2010 Sep 28;5(9):e12742. doi: 10.1371/journal.pone.0012742 (PMC2946934; doi:10.1371/journal.pone.0012742)
Supplement: Table S3 — NSHL X-linked, Y-linked and modifier loci. Locus names and chromosomal locations have been inferred from literature. References are relative to the articles where the locus association to NSHL was identified. (0.02 MB PDF) [file pone.0012742.s003.pdf]

Table S3. NSHL X-linked, Y-linked and modifier loci

LocusName	Position	References
DFN1	Xq22	[1]
DFN2	Xq22	[2]
DFN3	Xq21.1	[3]
DFN4	Xp21.2	[4]
DFN6	Xp22	[5]
DFNY1	Y	[6]
DFNM1	1q24	[7]

References

1. Tranebjaerg L, Schwartz C, Eriksen H, Andreasson S, Ponjavic V, et al. (1995) A new X linked recessive deafness syndrome with blindness, dystonia, fractures, and mental deficiency is linked to Xq22. J Med Genet 32: 257-263.

2. Tyson J, Bellman S, Newton V, Simpson P, Malcolm S, et al. (1996) Mapping of DFN2 to Xq22. Hum Mol Genet 5: 2055-2060.

3. Bach I, Robinson D, Thomas N, Ropers H, Cremers F (1992) Physical fine mapping of genes underlying X-linked deafness and non fra (X)-X-linked mental retardation at Xq21. Hum Genet 89: 620-624.

4. Lalwani A, Brister J, Fex J, Grundfast K, Pikus A, et al. (1994) A new nonsyndromic X-linked sensorineural hearing impairment linked to Xp21.2. Am J Hum Genet 55: 685-694.

5. del Castillo I, Villamar M, Sarduy M, Romero L, Herraiz C, et al. (1996) A novel locus for non-syndromic sensorineural deafness (DFN6) maps to chromosome Xp22. Hum Mol Genet 5: 1383-1387.

6. Wang Q, Lu C, Li N, Rao S, Shi Y, et al. (2004) Y-linked inheritance of non-syndromic hearing impairment in a large Chinese family. J Med Genet 41(6): e80.

7. Riazuddin S, Castelein C, Ahmed Z, Lalwani A, Mastroianni M, et al. (2000) Dominant modifier DFNM1 suppresses recessive deafness DFNB26. Nat Genet 26: 431-434.
